# A Novel Risk and Crisis Communication Platform to Bridge the Gap Between Policy Makers and the Public in the Context of the COVID-19 Crisis (PubliCo): Protocol for a Mixed Methods Study

**DOI:** 10.2196/33653

**Published:** 2021-11-01

**Authors:** Giovanni Spitale, Sonja Merten, Kristen Jafflin, Bettina Schwind, Andrea Kaiser-Grolimund, Nikola Biller-Andorno

**Affiliations:** 1 Institute of Biomedical Ethics and History of Medicine University of Zurich Zurich Switzerland; 2 Swiss Tropical and Public Health Institute Basel Switzerland; 3 University of Basel Basel Switzerland

**Keywords:** disease outbreaks, coronavirus, COVID-19 surveys, COVID-19 questionnaires, qualitative methods, health literacy, policy making, risk and crisis communication, COVID-19

## Abstract

**Background:**

Since the end of 2019, COVID-19 has had a significant impact on people around the globe. As governments institute more restrictive measures, public adherence could decrease and discontent may grow. Providing high-quality information and countering fake news are important. However, we also need feedback loops so that government officials can refine preventive measures and communication strategies. Policy makers need information—preferably based on real-time data—on people’s cognitive, emotional, and behavioral reactions to public health messages and restrictive measures. PubliCo aims to foster effective and tailored risk and crisis communication as well as provide an assessment of the risks and benefits of prevention and control measures, since their effectiveness depends on public trust and cooperation.

**Objective:**

Our project aims to develop a tool that helps tackle the COVID-19 infodemic, with a focus on enabling a nuanced and in-depth understanding of public perception. The project adopts a transdisciplinary multistakeholder approach, including participatory citizen science.

**Methods:**

We aim to combine a literature and media review and analysis as well as empirical research using mixed methods, including an online survey and diary-based research, both of which are ongoing and continuously updated. Building on real-time data and continuous data collection, our research results will be highly adaptable to the evolving situation.

**Results:**

As of September 2021, two-thirds of the proposed tool is operational. The current development cycles are focusing on analytics, user experience, and interface refinement. We have collected a total of 473 responses through PubliCo Survey and 22 diaries through PubliCo Diaries.

**Conclusions:**

Pilot data show that PubliCo is a promising and efficient concept for bidirectional risk and crisis communication in the context of public health crises. Further data are needed to assess its function at a larger scale or in the context of an issue other than COVID-19.

**International Registered Report Identifier (IRRID):**

DERR1-10.2196/33653

## Introduction

### Background

Since the end of 2019, COVID-19 has significantly impacted the lives of people around the globe. In addition to infection, disease, and death, the global public has been exposed to increasingly restrictive policy measures. Within weeks or even days, measures evolved from recommendations, such as frequent handwashing, to more disruptive interventions, including social distancing, cancelations of social events, closure of schools, and closed borders. Public life and ways of socializing that were once taken for granted have come to an abrupt halt.

Exceptional circumstances, like this pandemic, generally have significant short-, mid-, and long-term consequences in social, economic, and perhaps cultural and political terms. Some issues have already emerged, including social isolation of vulnerable groups, panic buying and stolen supplies, or instances of reprimanding people for their “irresponsible” behavior. While the gradual easing of containment measures alleviated frustration in parts of the population following the first wave, the reinstallment of restrictive measures may lead to mounting discontent and decreasing public adherence to containment measures.

Measures in Switzerland have been less restrictive than in many other countries. However, more drastic dispositions could be implemented and are legally covered by the Swiss Epidemics Law should the situation require them, including a general curfew, mandatory testing, or the use of mobile phone data for surveillance purposes. During the first wave (March to June 2020), the Swiss population generally supported the measures that were implemented. As subsequent waves unfold, however, debate on public health measures like contact tracing, limits on visiting nursing home residents, working from home, etc, has intensified.

“Anticorona” demonstrations in several cities, gatherings of hundreds of people celebrating the end of the lockdown, and organized “illegal” soccer games were among the first signs of resistance to public health measures in Switzerland [1]. In order to effectively manage the current pandemic crisis, we must better understand how the Swiss public perceives the public health measures implemented and concerns they have about the pandemic and the government’s response to it.

### Information Gaps

While governments are trying to steer through this crisis as cautiously as possible, the public is struggling to understand the situation. Communication is therefore key. Existing literature suggests that effective health communication can help enhance positive outcomes of public policy [2,3]. Importantly, exposure to focused health campaigns in the context of epidemics has proven to be an efficient tool not only to increase epidemic-related knowledge, but also to foster the adoption of recommended health behaviors [4,5].

While international organizations, national governments, public health authorities, scientific institutions, and high-quality media are trying to inform the public as responsibly as possible, many other information sources of questionable credibility exist across media platforms throughout Europe. Formal and informal opinion groups share content from these sources and influence public opinions in problematic ways, for example, by blaming specific social and ethnic groups for the pandemic or by encouraging defiance of public health recommendations. Some media draw on dystopic imagery and morally loaded language, using metaphors of war and reproaching those who voice doubts and criticism, which leads to polarization and affectively charged debates producing strong counterreactions rather than factual and nuanced public deliberation [6]. This situation has led the World Health Organization (WHO) to warn of an “infodemic,” wherein too much information of mixed quality make it difficult for people to find reliable information [7]. The WHO and other public health agencies are working on refuting myths regarding, for example, false preventive measures and false cures, through fact checks of social media and writing of responses [8].

However, providing high-quality information and countering fake news are not enough. Policy makers also need feedback loops to give them real-time data on people’s cognitive, emotional, and behavioral reactions to public health measures, allowing them to continuously refine and adjust preventive, control, and containment measures and communication strategies.

A better understanding of the population’s reaction to mitigation measures would allow for a better estimation of their potential effectiveness, influencing both communication strategies and policy choices [9,10]. It would also help to understand to what extent policy decisions match with citizens’ moral values and preferences regarding, for example, the allocation of scarce medical resources, contact tracing, or obligatory mask wearing [11]. Finally, understanding how different segments of the population perceive both the pandemic and public health measures is vital, as both disproportionately affected social groups that were already vulnerable before the pandemic, such as migrants and low-income workers [12]. How do, for example, frontline health care workers, older people, those who are chronically ill, or those who are economically vulnerable cope with the pandemic and mitigation measures? Given the limitations of “one-size-fits-all” approaches to mitigation measures, local and subgroup data are critically needed to develop more efficient strategies [13].

So far, there has been mainly “one-way communication.” We know little about different subgroups’ understanding of the situation and readiness to comply with policies, and how this is affected by their preferred sources of information. Cross-sectional opinion polls [14-16] encounter important limits in rapidly evolving situations—they are resource-intensive and limited in scope, their items are typically designed in a top-down way, and they struggle with high nonresponse rates and provide snapshots rather than continuous monitoring [11]. Consequently, policy makers might rely on a suboptimal picture of reality in order to make their choices, and some citizens may feel that large demonstrations are the only way to make themselves heard. Even if the majority of the public supports public policies and cooperates with them, this consensus may become fragile in the future if authorities disregard misunderstandings, concerns, or unrest in certain segments of the population. Better monitoring of public perceptions would enable better communication and more effective containment measures that reduce collateral damage to society.

However, such monitoring must be done in a way that citizens do not perceive as unwanted surveillance but rather as an initiative that invites their active input and values their views and opinions.

### Aims

PubliCo seeks to address these gaps. It is an experimental online platform built with a strong participatory citizen science component that will serve three purposes:

1. Collect real-time data on COVID-19–related public perception;
2. Provide tailored, timely, and reliable information to the public;
3. Facilitate well-targeted health policy making based on the theory that successful communication, public understanding, and consent reinforce the effectiveness of public health measures [2,3,5].

## Methods

### Concept

**Figure 1:**
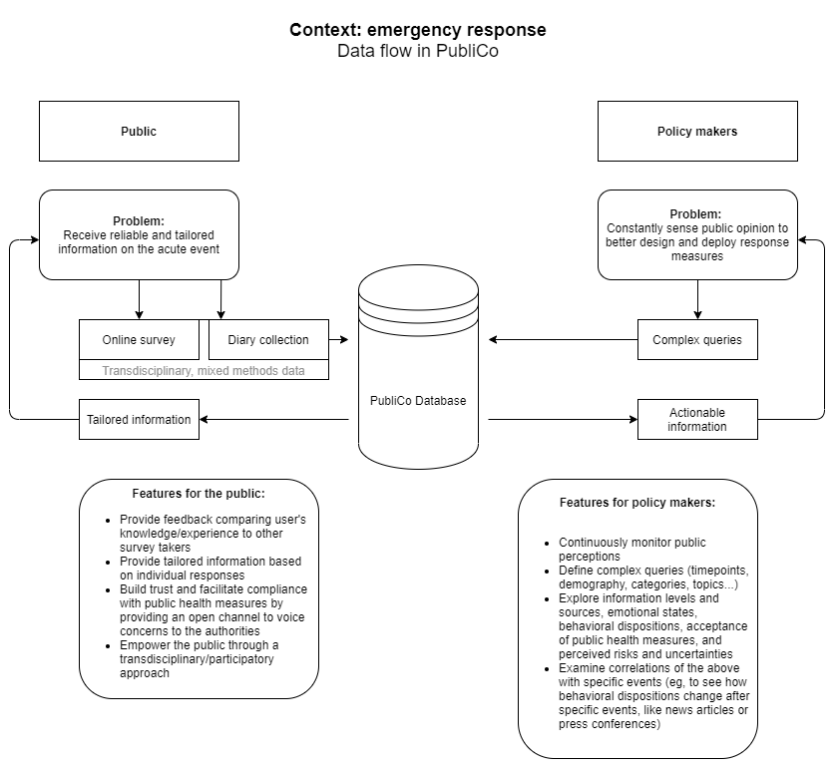
The PubliCo conceptual structure. After completing a short survey (PubliCo Survey), citizens can receive information tailored to their needs. Users can also register as citizen scientists and contribute diaries (PubliCo Diaries). Policy makers can study the information provided by citizens in order to conceive, deploy, and evaluate more efficient mitigation and containment measures (PubliCo Analytics).

The project combines analytical work and empirical studies using mixed methods and strong citizen science components in order to deliver a functional platform composed of three main elements: PubliCo Survey, PubliCo Diaries, and PubliCo Analytics (Figure 1).

PubliCo Survey will be the main source of quantitative information. Based on demographic characteristics and scores on selected subscales, citizens will obtain information specific to their needs. For example, people living in border regions will receive information about neighboring countries, and people with children will receive information about safety measures in schools. The survey will be ongoing, providing real-time data on public perception and readiness to cooperate with public health strategies.

PubliCo Diaries will be the main source of qualitative information. Qualitative solicited diaries can provide “unique insights into the life-worlds inhabited by individuals; their experiences, actions, behaviors, and emotions and how these are played out across time and space” [17]. The diary approach empowers citizens to integrate their personal experiences and perceptions [18] while remaining in control not only of the content described but also of the pace and time of data collection [17]. In this way, this participatory method allows for the involvement of citizens in the research process and the visualization of everyday negotiation processes in real time due to the immediacy of documentation [17,19].

Users will register as citizen scientists and keep a weekly diary to record their reflections on how COVID-19 and related policy measures affect their daily routine, social practices, values, and priorities. Citizen scientists may also keep their diaries offline or record audio files and have the text entered by project staff afterward so that segments of the population that do not have time to keep a written diary or are less tech-savvy can participate. In this way, PubliCo Diaries attempts to reach diverse groups of citizens currently encountering different personal situations and possibilities (eg, pregnant women, older people, people on short-time work, youth, or people with a migration background). These texts will provide information about meaning, as well as new insights on emerging, unforeseen impacts of the pandemic that diary authors discuss in their entries. Finally, qualitative analysis of the diary data will inform the revision or generation of new survey items.

PubliCo Analytics will be the “access door” to the data collected through the survey and the diaries. It will provide information to be used for analyses directed to policy makers regarding information levels, behavioral dispositions, emotional states, and moral preferences related to pandemic response measures. It also allows for analysis of correlations (eg, vaccine prioritization preference vs demographic subgroups; support of preventive measures vs COVID-19 experience). Finally, PubliCo Analytics will contain thematically focused policy briefs, in which we contextualize the data, interpret core findings, and make recommendations.

### Ethics Approval

As assessed by the Cantonal Ethics Committee of Canton Zurich, PubliCo does not fall under the scope of the Swiss Human Research Act (BASEC #2020-02917; December 15, 2020). Our risk assessment and data protection plan were also reviewed and approved by Ethics Review (CEBES), the institutional review board of the Institute of Biomedical Ethics and History of Medicine at the University of Zurich (CEBES #2020-13, December 15, 2020).

### Development

Developing the PubliCo platform involved work on three components:

- Development of PubliCo Survey and user feedback;
- Realization and testing of the platform;
- Definition of the analytic capabilities of PubliCo Analytics.

#### PubliCo Survey and User Feedback

In order to define the content of the survey and user feedback, we adopted a 3-fold strategy: identify the type of information people look for by analyzing Google Trends data, map the information available on media platforms through natural language processing (NLP) of news from major media outlets, and determine the focus of COVID-19–related behavioral and social science research (BSSR) assessing the content of the data collection instruments for COVID-19 compiled by the National Institutes of Health (NIH) Office of Behavioural and Social Sciences.

The analysis of Google Trends data on searches related to COVID-19 performed in Switzerland between January and July 2020 displayed great diversity in information consumption patterns; this varied greatly depending on the canton of residency. Swiss residents may therefore welcome a system like PubliCo, which delivers personalized information [20].

We identified the following categories of queries regarding the pandemic and its effects: georeferenced information, information from official sources (eg, WHO, federal authorities), quantitative information, news and updates, medical information, and tips.

In order to understand how the media discuss and frame COVID-19 in Switzerland, we used Factiva, a news-monitoring and search engine tool developed and owned by Dow Jones & Company that has access to full-text articles published by major media outlets worldwide. We gathered and downloaded all the news articles published between January and July 2020 on COVID-19 and Switzerland.

NLP and analysis of the frequencies of lemmas [21] revealed some differences across languages. The analysis of German lemmas indicates that public discourse was focused on the quantitative aspects of the pandemic. The French subcorpus focused on describing the pandemic and its effects on people. The Italian subcorpus focused more on cases and fatalities. The English subcorpus seemed to be dominated by information reported from other sources, which is expected since English is not an official language of the Confederation. It also contained many lemmas like “company,” “group,” and “market,” suggesting greater attention to the economic and financial impact of the pandemic [20].

All the subcorpora provided the following macrocategories of information: georeferenced information (information specific to countries, cantons, or cities); general information about the pandemic and the virus; reports from authorities and official bodies; and quantitative information.

The NIH Office of Behavioural and Social Sciences released a document listing “data collection instruments, including surveys, for assessing COVID-19-relevant BSSR domains for clinical or population research” [22]. Reviewing the surveys listed in the document, we identified 6 main topics of interest: financial impact, social practices, behavioral dispositions, moral preferences, emotional state, and cognitive understanding [20].

A comparison between information consumption patterns, information available in the media, and BSSR research interests identified 5 categories of information to collect and to provide through PubliCo: demographics, cognitive understanding, behavioral dispositions, emotional state, and moral orientations.

Citizen scientists will be involved in the validation of the survey and of the information we intend to provide. This will be accomplished through the web-based project builder of the Citizen Science Center Zurich [23].

#### Realization and Testing of the Platform

The PubliCo platform is being developed in cooperation with Belka, a software company based in Trento, Italy, and Munich, Germany, with extensive expertise in user experience design and development. The platform is web-based, mobile first, and built on a stack of open-source software: React (Facebook Open Source), SurveyJS (Devsoft Baltic), Typescript (Microsoft Corp), Diango (Django Software Foundation), MariaDB (MariaDB Foundation), Docker (Docker), CircleCI (Circle Internet Services), and NGINX (F5 Networks).

Particular attention is being devoted to the development of PubliCo Diaries, the interface through which registered citizen scientists can contribute their diaries. Early users have been involved in providing bottom-up feedback to refine and improve the interface. User experience testing will help ensure the platform is accessible to a large part of the Swiss population.

Another critical activity on the platform is the development of a backend for researchers, allowing nontechnical staff to view, add, and modify surveys, information for users, translations, and analytics components in an intuitive and collaborative way. The content management system fully supports a multilingual interface. Therefore, the final aim is to develop a tool that can be easily deployed and maintained everywhere, with little or no knowledge of the code running behind the interfaces.

#### Defining the Analytic Capabilities of PubliCo Analytics

Results from the online survey will be analyzed in multiple ways. Users will have direct feedback for certain variables (eg, information level, behavioral dispositions), including scores and official information based on responses to knowledge questions as well as basic descriptive statistics (means and frequencies) for all users and specific subgroups or respondents from specific cantons.

In addition, through PubliCo Analytics, researchers and policy makers will be able to answer complex questions like “Are people who know someone who got infected with COVID-19 more likely to get vaccinated?” and “How would people who have personal experience with COVID-19 prefer the vaccine to be distributed?” Queries can be restricted to specific subgroups (eg, age, residency, level of education).

Project researchers will also analyze results for periodic policy briefs. Questions to be examined will vary over time and will include basic descriptive statistics for the different domains included in the survey (knowledge, emotional state, behavioral dispositions, and moral preferences), subgroup analyses by geographical area and target group, and correlation analyses. Questions to be examined through the correlation analysis include:

- What is the relationship between participants’ knowledge and willingness to comply with public health restrictions?
- What is the relationship between participants’ knowledge and emotional state?
- What is the relationship between participants’ emotional state and their willingness to comply with public health restrictions?
- What factors influence participants’ moral preferences?

These and other questions will be analyzed using regression analysis with a significance level of α=.05.

The diary narratives will be anonymized and analyzed in conjunction with the ongoing data collection by means of thematic analysis [19] using the software MAXQDA (VERBI GmbH) [24].

**Figure 2:**
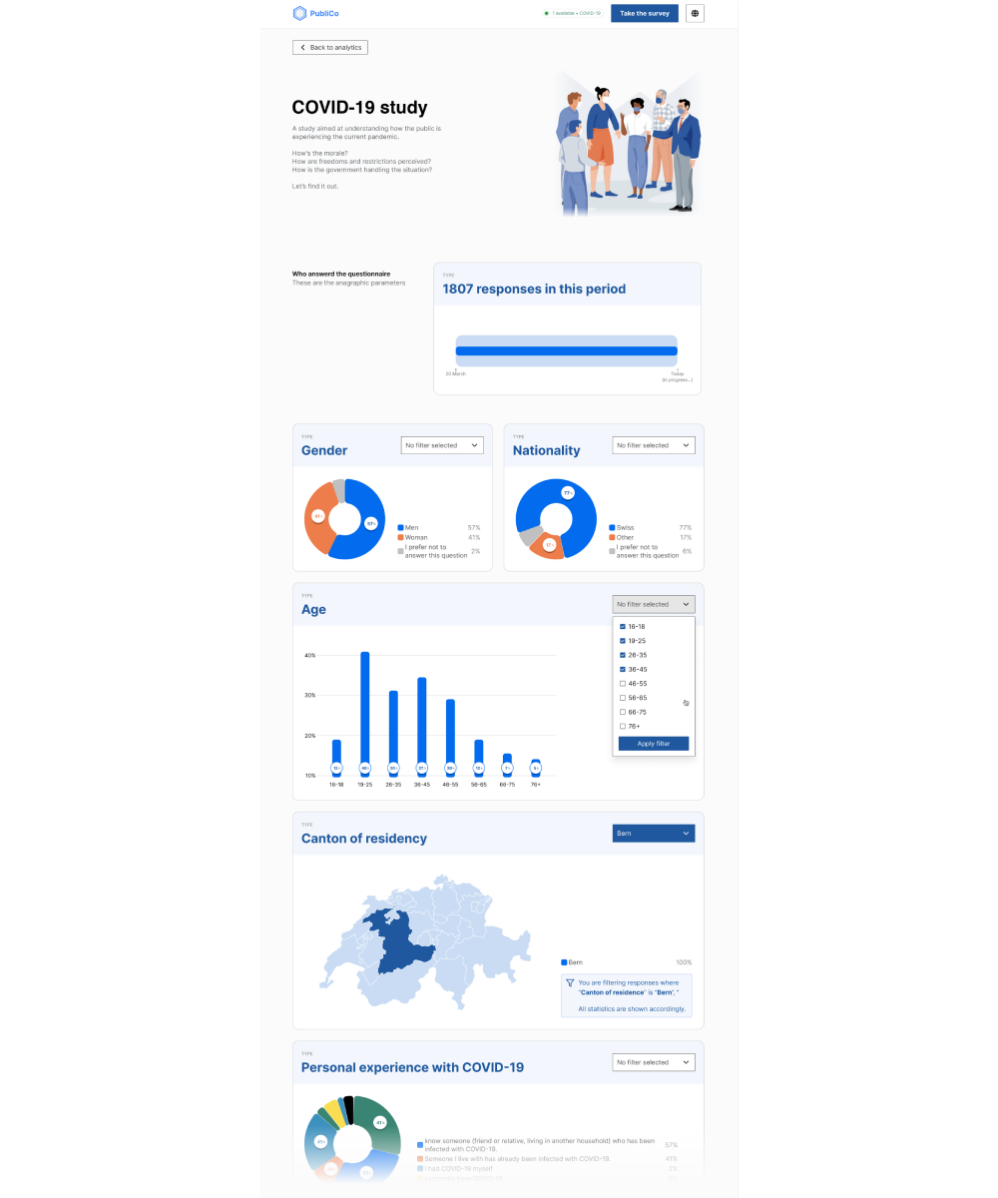
A high-level mock-up of PubliCo Analytics. Different kinds of survey data can be presented with an appropriate visualization. Visualizations can also be used to dynamically select a subset of the data frame (eg, selecting only specific demographic variables). The interface is meant to be informative, clear, and comprehensive to the general public. Every visualization is accompanied by an explanatory note.

Selected data will be displayed in PubliCo Analytics in a visually appealing way (eg, infographics, live maps), as shown in Figure 2 (for a higher-resolution version, see Multimedia Appendix 1).

Advanced analytics will be employed whenever possible (NLP for text elements; predictive modeling of, for example, public behavior in case of new measures implemented). Many passages, from the analysis of diaries to the automated analysis of selected subscales, will be automatized by means of NLP and other related artificial intelligence applications. These techniques will ensure that the platform is more cost-effective and that the results of the analysis and actionable information are available faster.

Data collection will be adapted to how the situation evolves, taking up emerging themes (eg, vaccine distribution, balancing work requirements, and protection of at-risk persons). Core findings and recommendations will be published in thematically focused policy briefs.

## Results

### Data Collection

Data collection for PubliCo Survey started with a pilot phase (December 2020 to April 2021), during which we collected analytics on how the platform and its different tools are used. For this purpose, we used a shorter version of the PubliCo survey, evaluated by citizen scientists through Citizen Science Center Zurich. This yielded more bottom-up input before deploying the full survey.

Data collection for PubliCo Diaries started during the pilot phase as well. Participants were given a brief guide to the diary method, which informed them about the openness of the method (eg, without concerns about spelling and grammar). The guide asked them to jot down their experiences and thoughts from the beginning of the pandemic to the current day and their everyday worries, emotions, risks, experiences, decisions, and actions during and/or after the pandemic on at least a weekly basis for a duration of at least 4 weeks. This will allow us to monitor changes in participants’ values, attitudes, level of knowledge, and behaviors [25].

Following the pilot phase, in order to increase the user base, PubliCo is in the process of being disseminated through:

- General media through featured articles in order to reach the general population;
- Mailing lists of the University of Zurich and the University of Basel in order to reach undergraduate and graduate students;
- Facebook groups in order to reach selected target groups, including migrants and parents;
- Teachers’ associations in order to reach high school students;
- Participants of the Swiss branch of the DIPEx International Study on COVID-19 in order to reach people who had direct experience of COVID-19;
- A demoscopic company that will solicit a representative sample for comparative purposes.

The outboarding section also invites the users to share the tool further via social media, email, or similar systems, and to register as citizen scientists for the PubliCo Diaries component. We will also investigate possibilities of disseminating through official channels, like the automatic SMS sender of the Federal Office of Public Health.

As of September 2021, we collected a total of 473 responses through PubliCo Survey, and 22 diaries through PubliCo Diaries. Data collection will be iterative and will proceed for at least 2 years. We expect the tool to be refined and enhanced as data collection and analysis moves forward. Because of the design of the tool, data saturation will be determined a posteriori by analyzing the demographic data of surveys and diary users. The current version of the tool is available at online [26].

### Availability of Data

#### Preliminary and Intermediate Data

The Google Trends data set used to define the survey component is available through our Zenodo repository [27]. The software used for the analysis of the Factiva corpus is also available through our Zenodo repository [28], as are the raw results of the analysis of the Factiva corpus [21]. Due to copyright restrictions, the Factiva corpus is available through Factiva.

#### Research Data

Data generated from PubliCo will be available through the PubliCo Analytics interface. Diary data are available upon request.

## Discussion

### Ethics and Dissemination

One aim of PubliCo is to deliver personalized information in the context of public health emergencies. However, providing personalized information can be potentially problematic. Feedback on knowledge-based questions simply involves notifying users of wrong answers and providing access to reliable sources, like the WHO or official information outlets [29]. Some uneasiness remains around making assumptions about citizens’ informational needs and possibly contributing to knowledge “bubbles.” Providing personalized information from subscales regarding emotional response, moral preferences, or mental well-being is more challenging. For these topics, we will provide a comparison between individual scores and sample means. In this sense, it is fundamental to clarify the descriptive nature of the scores without any claims as to what the norm should be (the is-ought problem). The final strategy needs to be defined with expert advisors and citizen scientists after evaluating potential outcomes.

The Swiss cantons have been affected in different ways by the COVID-19 pandemic. Our approach, comparing geolocated data, might reveal differences in behaviors and attitudes that could correlate with the course and the severity of the pandemic. Because of this, we will collect some demographic information (personal data; potentially also sensitive data as defined in the Law on Information and Data Protection (IDG) paragraph 3 of the Canton of Zurich) and some information about personal philosophical or religious beliefs (sensitive data as defined in IDG paragraph 3).

The potential harms generated by the project, assessed in Table 1, fall into two categories: reidentification (and thus attribution of specific opinions to specific persons) and morally problematic questions.

The most prominent category of risk is connected to the reidentification of participants. To minimize the chances of this, the survey component is completely anonymous by design (not even the IP [Internet Protocol] address is collected), and the diary component is pseudonymous (we can attribute diaries to users, but we cannot attribute users to persons). The only remaining concrete risk for reidentification is posed by what users could write in the diaries. Because of this, we are taking extra care in planning the access, use, and management of this category of data: no personal identifiers are collected upon registration, diary text is accessible upon request to trusted third parties (eg, research institutions), and the content is manually checked for full anonymity beforehand. We are confident that the instrument is safe from a data protection point of view.

All the data will be stored in a virtual machine hosted in the data center of the University of Zurich with access restricted to the project members. The chances of identification, in the eventuality of a data leak, are very low.

In order to mitigate the second category of risk, we are discussing the whole survey tool with expert advisors and citizen scientists in order to get additional feedback on the issues involved. However, the impact would still be low, and, more importantly, an unsatisfied user can pause or end participation at any time.

The very nature of this project implies another general risk: in a less democratic context, the tool we are developing could be used for social control. This is a potential risk we cannot mitigate for other countries. For Switzerland, the whole infrastructure of the project was built keeping in mind a transparent and democratic approach, important in general in the scientific enterprise, but fundamental in a context in which the data yielded from the system are used in order to make decisions impacting the public.

Overall, participants do not have an immediate personal benefit beyond the insights gained through the survey experience and feedback, but they do have a long-term community benefit resulting from the tool being used to deploy public health measures that consider and take into account their preferences. Therefore, we consider the risk-benefit balance justifiable.

**Table 1:** Risk assessment of PubliCo.

Potential event and consequences		Type of harm	Severity (1-5)	Likelihood (1-5)
**Reidentification of a participant**				
	Participants can feel betrayed by the data controller and lose trust in the research or society	Psychological	2	1
	Participants with controversial opinions could lose their jobs if these views are considered particularly dangerous by their employers	Economical	3	1
	Participants with controversial opinions could be rejected and isolated from the societies they belong to	Social	3	1
	Participants with controversial opinions could be physically assaulted because of their opinions	Physical	5	1
**Morally problematic questions**				
	Participants can be upset when asked about morally problematic topics (eg, allocation of scarce resources), especially if directly impacted by the issue at stake	Psychological	2	3

### Open Science by Design

We believe that adopting a democratic, bottom-up approach to design and develop PubliCo would greatly improve public perception of the project, while allowing us to tackle urgent and unforeseen issues [30]. As such, every component of PubliCo will be publicly available: the research project, the intermediate data sets and the software used to compile them, the source code, the raw data, and the interpretative briefs. The only data that will be subject to manual checks before release is the raw text of the diaries, as stated above.

This setup will increase trust in the project, encourage secondary use of PubliCo data, and facilitate the implementation of the tool in other countries.

### Limitations

This design has two main limitations. Our approach focuses on public perception rather than on observational data of real practices. There may be discrepancies between opinions, attitudes, and behavioral dispositions and what people do in reality. On the other hand, we think much insight is to be gained already from what people are, in principle, agreeable to or what they will consider unacceptable.

The second limitation concerns the information that is provided at the end of the survey. For some topics (eg, the concrete risk posed by COVID-19), it remains difficult to find solid metrics, and the way they are communicated can generate problems and misunderstandings. In this sense, we have opted to use a different approach: users will be pointed first to the official information provided by the Federal Office of Public Health, and secondly (depending on their scores in cognitive understanding) to PubMed queries designed to yield systematic reviews or meta-analyses. This way, following once again an open-science spirit, citizens will be able to access the relevant literature.

### Conclusions

Pilot data show that PubliCo is a promising and efficient concept for bidirectional risk and crisis communication in the context of public health crises, as it can reach and engage different segments of the Swiss population, collecting and providing information at the same time. Further data are needed to assess its function at a larger scale or in the context of an issue other than COVID-19.

## Appendix

Multimedia Appendix 1 Higher-resolution version of the high-level mock-up of PubliCo Analytics.

Multimedia Appendix 2 Peer-review report 1 by the Swiss National Science Foundation.

Multimedia Appendix 3 Peer-review report 2 by the Swiss National Science Foundation.

Multimedia Appendix 4 Peer-review report 3 by the Swiss National Science Foundation.

Multimedia Appendix 5 Peer-review report 4 by the Swiss National Science Foundation.

Multimedia Appendix 6 Peer-review report 5 by the Swiss National Science Foundation.

## Abbreviations

BSSR: behavioral and social science research
CEBES: Checkliste für den Ethik-Begutachtungsprozess von nichtbewilligungspflichtigen empirischen Studien (Ethics Review)
IDG: Law on Information and Data Protection
IP: Internet Protocol
NIH: National Institutes of Health
NLP: natural language processing
WHO: World Health Organization
