# Supplementary material for: A Novel Risk and Crisis Communication Platform to Bridge the Gap Between Policy Makers and the Public in the Context of the COVID-19 Crisis (PubliCo): Protocol for a Mixed Methods Study
Source: JMIR Res Protoc. 2021 Nov 1;10(11):e33653. doi: 10.2196/33653 (PMC8562419; doi:10.2196/33653)
Supplement: Multimedia Appendix 2 [file resprot_v10i11e33653_app2.pdf]

Review: 1

Application data

Applicant(s)
Biller-Andorno, Nikola
Merten, Sonja

PubliCo – an experimental online platform for COVID-19 related public perception
Special Call on Coronaviruses

Detailed evaluation

Scientific quality of the proposed research project

Overall, this was an excellent research proposal with a clearly articulated need. Some concerns exist about the ability to monitor impact

Specific strengths
- well qualified research team who have lead research in key areas
- the overall argument for the need for this platform (ie misinformation) is very convincing

Specific weaknesses
- I am not convinced that one post-doc is enough to deliver a usable project with the large range of outcomes stated
- General lack of clarity around the digital platform; what it will look like and how it will be ensured that it is usable and effective
- no evaluation of the effectiveness / impact of the platform.
- some concerns about the cost of the platform.

Qualification of the applicant(s)

Overall, the lead applicant is clearly a very successful scientist who leads research in a number of relevant areas. In particular previous expertise on 'digital support of patient healthcare decisions' looks relevant.

Specific strengths
As above - a well established researcher with a relevant and high-profile track record.

Specific weaknesses
- I do have some concerns that one postdoc (only working for 15% in year 2) would be enough to deliver a lot of this work, although the applicants list a large and very experienced core research team who could be expected to do some of the work

Alignment of the application to the identified call priority areas

Well aligned with the call. The research team clearly has a lot of expertise regarding the 'ethical considerations' call in particular but this research will contribute a lot to societal impact.

Specific strengths
An important and relevant piece of work.

Specific weaknesses
None

Potential for timely and significant contributions to the research field

Generally the potential for impact is good - although i could not clearly see how this would be evaluated. This is my largest concern for this project; that the ambition is large and the platform development and optimisation may take longer than initally planned. I would also have liked to see a clearer pathway of how the website will be disseminated to influence policy *during* this pandemic, rather than for future pandemics.

Specific strengths
Potential for contribution is large.

Specific weaknesses
- I do have some concerns about the timing of this project. the initial development of content and website development look to be done incredibly quickly.
- the literature reviews, if done sysematically, and the media analysis may take a substantial amount of time. Are these going to be conducted before the development of the platform or during? If during/after, are there sufficient costs to cover the development costs? these can rack up quickly.
- how exactly will they be analysed? will a framework for future analysis be developed?
- regarding development costs... this platform could be quite substantial and will need piloting extensively. i would implore the applicants and board to make absolutely clear that they have costed enough for this!
- I would like to see much more clarity about how the extensive stakeholder list, and the citizen scientists, will provide feedback and input to the devwelopment of the tools. In my experience this iterative optimisation approach can take some time and a clearly structured approach is required to make it sufficiently rapid.
- will

Financial Request

Comment

- a very well thought out project with clear and useful goals
- some concerns about the ambition and whether an effective platform can be created within the time and budget (perhaps allocate more budget to the development if required)
- I am not sure the stated goal of evaluating impact is going to be achieved.

Note on the evaluation procedure

The proposals have been evaluated by members of an international pool of experts, most of whom reviewed several proposals. As outlined in the call document, proposals were graded and ranked based on the assessments by the experts. The decision was approved by the Presiding Board of the Research Council of the Swiss National Science Foundation.
