# Supplementary material for: A Novel Risk and Crisis Communication Platform to Bridge the Gap Between Policy Makers and the Public in the Context of the COVID-19 Crisis (PubliCo): Protocol for a Mixed Methods Study
Source: JMIR Res Protoc. 2021 Nov 1;10(11):e33653. doi: 10.2196/33653 (PMC8562419; doi:10.2196/33653)
Supplement: Multimedia Appendix 3 [file resprot_v10i11e33653_app3.pdf]

Review: 2

Application data

Applicant(s)

Biller-Andorno, Nikola

Merten, Sonja

PubliCo – an experimental online platform for COVID-19 related public perception

Special Call on Coronaviruses

Detailed evaluation

Scientific quality of the proposed research project

Covid 19 has created many of the expected communication challenges including high uncertainty, high threat and the need to move quickly. The scope and scale of this pandemic has accentuated many of those conditions.

Specific strengths
Investigating the public perceptions of covid 19 will be critical to exciting appropriate public responses, including self protective behaviors.

Specific weaknesses
The project does now seem to be grounded in a clear theatrical framework limiting generalizability.

Qualification of the applicant(s)

The project is being run be established scholars with strong records of excellent work.

Specific strengths
The team has strong disciplinary and interdisciplinary backgrounds.

Specific weaknesses
The team could benefit from a member with a communication background.

Alignment of the application to the identified call priority areas

The project touches on several of the priority areas.

Specific strengths
The connection between the social media communications on understanding and behavior and the response systems and crisis management is important.

Specific weaknesses
The project might also examine the ways messages are developed by agencies and SMEs.

Potential for timely and significant contributions to the research field

Social science research generally takes time.

Specific strengths
The project has the potential to make especially important contributions within the context of a real event.

Specific weaknesses
The project needs to take into account the time dimensions as crisis events ands communication change very quickly.

Financial Request

No comment.

Comment

This is an important project that should yield important insights for both this and other public health emergencies.

Note on the evaluation procedure

The proposals have been evaluated by members of an international pool of experts, most of whom reviewed several proposals. As outlined in the call document, proposals were graded and ranked based on the assessments by the experts. The decision was approved by the Presiding Board of the Research Council of the Swiss National Science Foundation.
