# Supplementary material for: A Novel Risk and Crisis Communication Platform to Bridge the Gap Between Policy Makers and the Public in the Context of the COVID-19 Crisis (PubliCo): Protocol for a Mixed Methods Study
Source: JMIR Res Protoc. 2021 Nov 1;10(11):e33653. doi: 10.2196/33653 (PMC8562419; doi:10.2196/33653)
Supplement: Multimedia Appendix 4 [file resprot_v10i11e33653_app4.pdf]

Review: 3

Application data

Applicant(s)

Biller-Andorno, Nikola

Merten, Sonja

PubliCo – an experimental online platform for COVID-19 related public perception

Special Call on Coronaviruses

Detailed evaluation

Scientific quality of the proposed research project

Authors provide a clear and well-justified rationale for the proposal focusing on a nuanced and in-depth understanding of the public perception of various aspects related to the pandemic outbreak. The key focus in on 'crowdsourcing' public opinions using the custom-made online platform with a set of curated surveys conducted with individual citizens, as well as scraping specific online outlets and social media sites. Each step of this approach is well-justified by applicants: illustrating clear research gap (there is no comparable platform that provides such comprehensive resource of information and surveying capabilities) and clear methodology employed to achieve intended outcomes (media data collection, survey design related to emotional state, behavioural dispositions, moral preferences, approach to platform design). Employment of specific survey and data collection design is well-positioned within existing theoretical frameworks (e.g. Moral Machine approach for moral preference survey).

Specific strengths

It's a sophisticated attempt to aggregate, analyse and provide context around public perception and behaviour around pandemic and crisis. As such it has many pathways to impact that applicants clearly highlight - from practical usefulness for a policymaker to get an estimate of how the public perceives/responds to crisis, to a broad range of potential scientific insights obtained from rich data set collected as part of this project.

Specific weaknesses

Ensuring participation and avoiding attrition of involved stakeholders (citizen scientists, expert council, but primarily survey participants) is a potential weakness. Online participation in longitudinal projects is notorious for high attrition rates, and this may be reinforced by a relatively large number of surveys and diaries various participants will be requested to fill. Applicants are clearly aware of this challenge and outline this as potential risks in point B & D (page 10). This point may be particularly problematic for long-term maintenance of the platform as online attrition tends to increase over time.

This links with a larger question related to applicants plan to maintain PubliCo platform beyond the scope of the project. In general, such complex online platforms require continuous servicing and updates, as the dynamics of data sets and digital tools used to build the website evolves. To ensure the relevance and usability of the platform for any future crisis events, applicants should provide a brief plan about how they hope to maintain this platform after the project reaches completion.

There are also some questions around resourcing for data mining tasks - see Qualification section.

Qualification of the applicant(s)

Both applicants represent an excellent level of expertise and experience in specific fields relevant to the application topic - epidemiology, public health, medicine, interdisciplinary research.

Specific strengths
The main applicant has an extensive track of experience working in an interdisciplinary and practical approach to epidemiology - from field research to specific uptake evaluation. She has an excellent track record in project management based on past grants and achievements history. Co-applicant also has already been involved in a project around similar scope, namely "Harnessing the Power of Patient Narratives".

Specific weaknesses
There should be more evidence that applicants have sufficient human resources to handle large data mining and analytics operation related to this project. I see there is one person in the core team (Dr Kristen Jafflin) with this specific skill focus. From applicants description, it's clear that data analysis for this platform will likely to be an extensive and potentially labour intensive process. This will require a strategy for data analytics that will encompass areas such as (1) ongoing data scraping, management, storage and sharing procedures and associated data ethics issues (applicants have an excellent background in ethics so good practices are expected here by default), (2) data processing protocols (data will be coming from many different sources - news, social networks, surveys, diaries - data will also be of mixed quality; there has to be a clear processing pipeline to ensure integrity, validity and reliability of the collected data), (3) actual analytics for real-time, post-hoc, and infographic reporting (analytics output of this project will take many different forms - interactive real-time components and infographic on the platform website, reports to various stakeholders, thematic analysis, survey analysis with "automated descriptive and inferential statistics performed on selected subscales").

It seems that the idea is to delegate some of this work to the "core team" (a group of supporting researchers named on page 6) and the "citizen scientists" that applicants will recruit for the project. Some of it will be done by the company, which is sensible for the online content (but has limits - the company might require support for a specific type of analysis or data scraping process). Applicants stress that RA employed by the grant will have good quantitative/qualitative skills, although it seems this RA will be very busy with other administrational duties that are related to this project.

Overall, it would be advisable for applicants to have someone with good time availability working specifically in the data science/analysis/mining space of the project.

Alignment of the application to the identified call priority areas

Applicants proposal seems to align best with two priority areas: (1) "The impact of official and social media communications on understanding and behaviour of policymakers, health care workers, patients and populations and (2) "The societal impact at large, including developing strategies to understand and combat misinformation, stigma, and fear". For both priority areas, there is a clear illustration that proposal outcomes would support (1) by examining sentiments, behavioural dispositions, and moral preferences in sampled population and (2) providing online tools and reports for stakeholders dealing with crisis impact.

Specific strengths
-

Specific weaknesses
-

Potential for timely and significant contributions to the research field

There is a clear pathway in this proposal to impact via developing a potential model of how such online 'population attitudes sampling' platforms could be deployed in the future crisis. The rich dataset collected by applicants could be further analysed to test specific social and psychological hypotheses. See more feedback I've provided in the previous questions regarding contribution.

Specific strengths
-

Specific weaknesses
-

Financial Request

Comment

This is a well-justified idea to develop a comprehensive online platform used for the collection of public perceptions related to COVID-19 outbreak - with multiple sources of data and contextual information. Such platform could aid policymakers in their decisions during a crisis situation, could be used in training and games related to crisis management, and could provide unique opportunity to examine population response to such crisis from an emotional, behavioural and moral perspective. Applicants have an excellent level of expertise with relevant experience to create such an online platform. Two minor points to consider are (1) related to the longevity of the platform beyond the project scope (maintaining it for future applications and further research) and (2) related to data mining challenges and resource allocation for this task.

Note on the evaluation procedure

The proposals have been evaluated by members of an international pool of experts, most of whom reviewed several proposals. As outlined in the call document, proposals were graded and ranked based on the assessments by the experts. The decision was approved by the Presiding Board of the Research Council of the Swiss National Science Foundation.
