# Supplementary material for: A Novel Risk and Crisis Communication Platform to Bridge the Gap Between Policy Makers and the Public in the Context of the COVID-19 Crisis (PubliCo): Protocol for a Mixed Methods Study
Source: JMIR Res Protoc. 2021 Nov 1;10(11):e33653. doi: 10.2196/33653 (PMC8562419; doi:10.2196/33653)
Supplement: Multimedia Appendix 5 [file resprot_v10i11e33653_app5.pdf]

FONDS NATIONAL SUISSE
SCHWEIZERISCHER NATIONALFONDS
FONDO NAZIONALE SVIZZERO
SWISS NATIONAL SCIENCE FOUNDATION

Review: 4

Application data

Applicant(s)

Biller-Andorno, Nikola
Merten, Sonja

PubliCo – an experimental online platform for COVID-19 related public perception

Special Call on Coronaviruses

Detailed evaluation

Scientific quality of the proposed research project

This is an innovative and sophisticated project with potential to reveal public understandings of COVID19 (resulting from existing media/communication), their emotional/behavioural reactions thereto, and their moral preferences regarding policy development. Insights gained from this research will be valuable for crucial policy development with regard to risk and crises communication and in cases involving value trade-offs (for which knowledge of public preferences will be valuable).

Specific strengths

This proposal involves exceptionally well-developed and state of the art project design involving a transdisciplinary multi-stakeholder approach and mixed methodologies, for which the research team has extensive experience/expertise. The "key dimensions" outlined on p. 8 are especially innovative and insightful--and will yield a variety of significant findings that will feed into important policy recommendations (via a series of policy briefs).

Specific weaknesses

In various places the proposal suggests that the project might involve or do one thing or another--leaving some unclarity about what exactly will occur and/or should be expected. This is perhaps not a serious weakness and may, to the contrary, reflect flexibility, openness to possible opportunities, and the dynamic nature of the activities envisioned. Plenty of what is planned is (more than) sufficiently clear and concrete in any case.

Qualification of the applicant(s)

Both applicants have outstanding research track records--and their areas of expertise/experience are highly complementary and directly relevant to the proposed project.

Specific strengths

Both applicants have strong cvs and publication track records. Both hold senior leadership roles at their institutions. Both have impressive achievements with respect to being awarded grant funding and successfully conducting relevant research. Both have expertise and backgrounds that are particularly well-suited for the proposed project. Beyond PIs, this project will have an impressive "core team" of researchers with diverse backgrounds to draw on, as outlined on p 7.

Specific weaknesses

Though it is evident that the applicants have previously published together, it would have been better if the proposal said more about their past collaboration and/or ability to work together.

Alignment of the application to the identified call priority areas

This project's alignment with call priority areas is outstanding.

Specific strengths
This is a highly ambitious project. The applicants correctly note that it directly addresses 3 call priority areas:
-The societal impact at large, including developing strategies to understand and combat misinformation, stigma, and fear
-The impact of official and social media communications on understanding and behaviour of policy makers, health care workers, patients and populations
-Contribution to ethical considerations

Specific weaknesses
N/A

Potential for timely and significant contributions to the research field

This project has potential to make significant, timely contributions to public communication and other policy responses to COVID-19--and to strengthen public understanding, trust and compliance in the process. It thus has high potential to make concrete impacts.

Specific strengths
This project will provide timely findings regarding public perceptions of COVID1-19, including emotional states, behavioural dispositions, changes in social practices, and moral preferences. Thus filling an important gap in knowledge in the Swiss context, it will feed into policy making regarding risk and crisis communication and other aspects of policy making (that should be informed by findings regarding moral preferences in particular). Findings from this project--and the Publico tool developed--may also have application for other crises (in the future).

Specific weaknesses
It might have been better to provide more detail regarding plans for development of policy briefs and academic outputs.

Financial Request

The financial request appears reasonable to me--but my expertise for judging/assessing this is admittedly limited.

Comment

This is an outstanding and innovative project that promises to fill a gap in knowledge regarding public understandings of COVID-19 (resulting from existing modes of media communication), emotional/behavioural reactions thereto, and value preferences (relevant to policy making). By feeding these findings into policy making, it has potential to significantly improve relevant risk and crises communication, the making of policy that is aligned with public values--and ultimately improved public understanding, trust, and compliance with public health policy regarding COVID-19. The project design is exceptionally well developed and highly sophisticated, involving a transdisciplinary multi-stakeholder approach and mixed methodologies. The project is directly relevant to 3 call priority areas. Among other virtues, the findings of this research--and the online tool developed--may have valuable application to other (future) crises.

Note on the evaluation procedure

The proposals have been evaluated by members of an international pool of experts, most of whom reviewed several proposals. As outlined in the call document, proposals were graded and ranked based on the assessments by the experts. The decision was approved by the Presiding Board of the Research Council of the Swiss National Science Foundation.
