# Supplementary material for: A Novel Risk and Crisis Communication Platform to Bridge the Gap Between Policy Makers and the Public in the Context of the COVID-19 Crisis (PubliCo): Protocol for a Mixed Methods Study
Source: JMIR Res Protoc. 2021 Nov 1;10(11):e33653. doi: 10.2196/33653 (PMC8562419; doi:10.2196/33653)
Supplement: Multimedia Appendix 6 [file resprot_v10i11e33653_app6.pdf]

FONDS NATIONAL SUISSE
SCHWEIZERISCHER NATIONALFONDS
FONDO NAZIONALE SVIZZERO
SWISS NATIONAL SCIENCE FOUNDATION

Review: 5

Application data

Applicant(s)
Biller-Andorno, Nikola
Merten, Sonja

PubliCo – an experimental online platform for COVID-19 related public perception
Special Call on Coronaviruses

Detailed evaluation

Scientific quality of the proposed research project

The researchers have proposed developing a website for collecting information about how people have responded to the pandemic. This includes testing their knowledge and assessing their emotional states and behavioral decisions. The information collected with this website could be valuable for policymakers and researchers examining public response to the pandemic.

Specific strengths
This proposed website would assess many of the important facets of pandemic response: knowledge, emotional response, and behavioral decisions. Together, these factors provide a comprehensive understanding of public response.

Specific weaknesses
There are several weaknesses that may limit the scope of this project. The first is that it is unclear who exactly will be taking these surveys on the website. The researchers have explained "everyday" individuals, so to speak, will take these surveys. However, no information is given about how these people will be included or what incentives will be provided for participating in these surveys. The researchers have explained the procedure for the diary collection (and included information about possible financial incentives), but this information is not provided for the general website. Why would someone want to take the surveys? How will this website be publicized. Furthermore, the researchers specify that certain demographics would be valuable for examination including the elderly and those of lower socioeconomic status. However, these populations are often the ones with the most limited access to or ability to use online tools. How will the researchers ensure that these populations will be reached?

The second weakness is that it is unclear who will ultimately have access to the data (and how individuals will gain access). The researchers state that policymakers and other decision makers MIGHT have access to the back end data at a later date. This access is central to the argument for why this website could be valuable. Without being certain that this data will be available to those who could benefit from it, it is difficult to see how this project could benefit the general public.

Furthermore, it seems as if this data will not be available to the general public, but rather to those who are in positions of power and action. What does the general public ultimately have to gain from this website? Specifying how this will help "everyday" people is important.

Regarding methodology: the content analysis portion seems vital to understanding how individuals have gained the knowledge they have about the virus. However, by basing the sampling procedure on content individuals tell about, the researchers are severely limiting their understanding of what media and social media content includes. Individuals are not great about recalling content that they've encountered. As such, a great deal of content they've consumed will be left out of the analysis, making it difficult to draw conclusions about exposure effects.

Qualification of the applicant(s)

This proposal brings together a wide variety of individuals as part of the core team and the expert council.

Specific strengths
Having such a wide variety of individuals as part of both the core team and the expert council is a good idea because it will allow the main researchers to have input from a diverse group of experienced people. The main two researchers also have extensive research experience, as evidenced by their publication and grant history.

Specific weaknesses
During the past several years, both lead researchers have had limited first authored publications.

Alignment of the application to the identified call priority areas

The researchers have helpfully outlined which specific call priority areas their project addresses.

Specific strengths
The information gained from the development of the proposed website would certainly address the "understand" portion of "understand and combat misinformation, stigma, and fear." By testing knowledge of the virus, it would help the researchers know which information is sticking with people. The fear portion would be addressed by the emotional assessment.

Specific weaknesses
As I read it, this particular call priority area is aimed at understanding AND combating these issues. The website would collect information aimed at understanding, but it is unclear from the proposal how this information will be used to combat these issues. It seems that the website will not have much (if any) audience facing content aimed at providing information about the virus. If the researchers do ultimately allow non-researcher access to the data, it is unclear how this data will be used to combat these issues.

Potential for timely and significant contributions to the research field

The researchers have provided a realistic timeline for this project.

Specific strengths
The website would be up and running relatively quickly and could be adapted to shifts in knowledge and response to the pandemic.

Specific weaknesses
It seems that it would be an entire year before any conclusions could be drawn about what the public knows about the virus and their responses to it.

Financial Request

The researchers have requested exactly 300,000 Swiss francs.

Although it is within the typical request amount, there is one concern about the budget. The researchers have included a quote from the website designer that is well above their included budget for that portion of the work. They have stated that they believe it will ultimately be cheaper and within their 60,000 budget listing, but it is unclear how this will occur.

Comment

Although the qualifications of the researchers are very good, the limitations of the proposed research project and the alignment of the application to the identified call priority areas have averaged out to a "good" rating.

Note on the evaluation procedure

The proposals have been evaluated by members of an international pool of experts, most of whom reviewed several proposals. As outlined in the call document, proposals were graded and ranked based on the assessments by the experts. The decision was approved by the Presiding Board of the Research Council of the Swiss National Science Foundation.
